# Practical Guidance on Initiating and Switching Targeted Immunotherapies in Generalised Myasthenia Gravis: A German–Austrian Expert Opinion Paper

**DOI:** 10.1111/ene.70747

**Published:** 2026-09-09

**Authors:** Andreas Meisel, Adela Della Marina, Paolo Doksani, Tim Hagenacker, Christoph Kleinschnitz, Heidrun H. Krämer, Jan D. Lünemann, Sven G. Meuth, Tobias Ruck, Ulrike Schara‐Schmidt, Christiane Schneider‐Gold, Benedikt Schoser, Michael Schroeter, Charlotte Schubert, Jörn Peter Sieb, Fritz Zimprich, Jana Zschüntzsch, Sarah Hoffmann

**Affiliations:** ^1^ Department of Neurology With Experimental Neurology Charité – Universitätsmedizin Berlin, Corporate Member of Freie Universität Berlin and Humboldt‐Universität zu Berlin Berlin Germany; ^2^ Neuroscience Clinical Research Center Charité – Universitätsmedizin Berlin, Corporate Member of Freie Universität Berlin and Humboldt‐Universität zu Berlin Berlin Germany; ^3^ Charité – Universitätsmedizin Berlin, Corporate Member of Freie Universität Berlin and Humboldt‐Universität zu Berlin Berlin Germany; ^4^ Department of Pediatric Neurology, Center for Neuromuscular Disorders in Children and Adolescents University Hospital Essen, University Duisburg‐Essen Essen Germany; ^5^ Department of Neurology and Center for Translational Neuro‐ and Behavioral Sciences (C‐TNBS) University Hospital Essen Essen Germany; ^6^ Department of Neurology Justus‐Liebig‐University Giessen Giessen Germany; ^7^ Division of Neurology, Department of Medicine University of British Columbia Vancouver British Columbia Canada; ^8^ Djavad Mowafaghian Centre for Brain Health, University of British Columbia Vancouver British Columbia Canada; ^9^ School of Biomedical Engineering University of British Columbia Vancouver British Columbia Canada; ^10^ Department of Neurology University Hospital Münster Münster Germany; ^11^ Department of Neurology Heinrich‐Heine‐University Düsseldorf Düsseldorf Germany; ^12^ Department of Neurology Ruhr University Bochum, BG University Hospital Bergmannsheil, Heimer Institute for Muscle Research Bochum Germany; ^13^ Department of Neurology, St. Josef Hospital Ruhr‐University Bochum Bochum Germany; ^14^ Friedrich‐Baur‐Institut Neurologische Klinik und Poliklinik LMU Klinikum München, Friedrich‐Baur‐Institute, Department of Neurology LMU Clinics Munich Munich Germany; ^15^ Department of Neurology University of Cologne and University Hospital Cologne Germany; ^16^ Department of Neurology Institute of Neuroimmunology and MS (INIMS), University Medical Center Hamburg‐Eppendorf Hamburg Germany; ^17^ Department of Neurology Helios Hanseklinikum Stralsund Stralsund Germany; ^18^ Department of Neurology Medical University of Vienna Vienna Austria; ^19^ Department of Neurology University Medical Center Göttingen Göttingen Germany

**Keywords:** clinical practice, complement inhibitors, generalised myasthenia gravis, neonatal fc receptor inhibitors, targeted immunotherapies

## Abstract

**Background:**

The therapeutic landscape of generalised myasthenia gravis (gMG) has evolved substantially with the approval of targeted immunotherapies, including complement C5 inhibitors (C5‐I) and neonatal Fc receptor inhibitors (FcRn‐I). While pivotal trials have demonstrated marked efficacy in defined subgroups, real‐world experience reveals more heterogeneous outcomes and raises questions about optimal patient selection, therapy sequencing and integration into clinical practice. In Germany and Austria, early access following regulatory approval has facilitated clinical experience over recent years. This expert opinion paper aims to combine current evidence with clinical experience to guide the use of C5‐I and FcRn‐I in everyday care.

**Methods:**

A panel of 18 neurologists from Germany and Austria, including two paediatric neurologists, evaluated study data and real‐world experiences. Through structured discussion and a consensus process, they developed evidence‐ and experience‐based recommendations on integrating targeted immunotherapies into existing treatment algorithms.

**Results:**

The panel provides practical recommendations for managing (highly) active gMG in adults, focusing on C5‐I (eculizumab, ravulizumab, zilucoplan) and FcRn‐I (efgartigimod, rozanolixizumab). The statements cover initiation criteria, sequencing and switching within and between drug classes and transitions to intensified immunomodulatory therapies (rituximab, apheresis, intravenous or subcutaneous immunoglobulin), as well as special considerations for juvenile MG.

**Conclusions:**

This expert statement provides a practice‐oriented framework integrating current evidence and clinical experience to support individualised therapeutic decision‐making in gMG.

## 1 Introduction

The therapeutic landscape of generalised myasthenia gravis (gMG) has undergone a remarkable transformation in recent years. For decades, treatment options beyond symptomatic and broad immunosuppressive approaches were limited. Now, several pivotal phase 3 trials have demonstrated robust efficacy of targeted immunotherapies, leading to successive regulatory approvals [1, 2, 3, 4, 5, 6, 7]. Among these, complement C5 inhibitors (C5‐I) and neonatal Fc receptor inhibitors (FcRn‐I) have been most prominent, representing the first generation of targeted immunotherapies that intervene at downstream points of the autoimmune cascade by either reducing pathogenic antibody levels or blocking complement‐mediated effector mechanisms of acetylcholine receptor autoantibodies (AChR‐Ab).

While clinical trial data have confirmed significant benefits in defined patient populations, real‐world experience has revealed more heterogeneous outcomes, including variable magnitude and durability of clinical responses, relevant proportions of patients with insufficient benefit, and persistent occurrence of exacerbations or crises despite treatment [8, 9, 10, 11, 12, 13, 14]. Uncertainties remain concerning optimal timing of treatment initiation, management of non‐responders, as well as approaches to the mitigation of adverse events (AEs). In Germany and Austria, the prompt availability of newly approved therapies has enabled clinicians to gain early real‐world experience with C5‐I and FcRn‐I, providing a foundation for shared expert consensus and practical treatment guidance.

In this context, this expert opinion paper reflects the consensus of German and Austrian MG experts, integrating early real‐world experience and translating emerging evidence into practical recommendations for the initiation, sequencing, and switching of targeted immunotherapies in gMG. With a particular focus on C5‐I and FcRn‐I, it aims to guide clinicians through a rapidly evolving therapeutic landscape and to link clinical trial data with routine clinical practice.

## 2 Methods

This expert opinion paper aims to provide practice‐oriented recommendations for the use of targeted immunotherapies in gMG. The narrative text synthesises available evidence from clinical trials and real‐world studies, together with regulatory information and, where relevant, recommendations from the German guideline. The expert consensus statements focus on clinical scenarios in which evidence is limited or absent, including treatment initiation, sequencing, switching, treatment duration, de‐escalation, and management after rescue therapy. These statements were developed through structured expert discussion and formal voting, as described below. A panel of 18 neurologists (17 from Germany and one from Austria), including two paediatric neurologists, with extensive experience in gMG management, was convened. The lead authors, A. Meisel and S. Hoffmann, held a series of online meetings with the group of experts. Here, a methodological framework in accordance with established standards for consensus development and the main topics were defined and subsequently discussed further in parallel meetings by four therapy‐specific working groups focusing on (1) C5‐I, (2) FcRn‐I, (3) rituximab (RTX) and intravenous immunoglobulins (IVIg) as intensified immunomodulatory therapies and (4) management of juvenile MG (jMG). Nipocalimab and inebilizumab had not received European Medicines Agency (EMA) approval at the time of the expert meetings and were therefore not included in the expert consensus process or formal voting. To reflect the evolving therapeutic landscape, both agents are nevertheless briefly mentioned throughout the manuscript where appropriate.

Each working group meeting was recorded and the most important statements were summarised. An overarching summary and expert consensus statements were then developed by the lead authors and reviewed and discussed in two meetings within the full panel. The expert consensus statements were subjected to a formal voting process by the whole expert group. A recommendation was considered accepted if it achieved > 75% agreement (< 50%: no consensus; > 50%–75%: majority vote; > 75%–95%: consensus; > 95%: strong consensus). The expert consensus statements are highlighted in boxes as recommendations (‘Expert consensus’) and the degree of consensus is indicated in parentheses. Statements that did not require extensive discussion (i.e., label recommendations, vaccination recommendations) were adopted without a voting procedure and are also described. Recommendations were organised according to their clinical context and practical application rather than the degree of consensus. The paper was drafted by the principal authors, then reviewed and approved by all authors.

## 3 Results

The approval status (according to EMA) and the corresponding recommendations of the German guideline for the use of targeted immunotherapies in gMG are summarised in Table 1. In general, the authors align with the guidance provided in the latter [15]. Accordingly, the management of gMG should consider the patient's age, thymic pathology, antibody status (AChR‐, MuSK‐, LRP4‐antibody‐positive, or seronegative MG) and overall disease activity [15].

**TABLE 1:** Approval status (according to the EMA) and German guideline recommendations for use of C5‐I and FcRn‐I in (highly) active gMG [15, 16, 17, 18, 19, 20, 21, 22].

	Add‐on therapy to standard treatment for adults with							
	AChR‐Ab‐positive gMG		MuSK‐Ab‐positive gMG		LRP4‐Ab‐positive gMG		Seronegative gMG	
	APR	GGR	APR	GGR	APR	GGR	APR	GGR
C5‐I	√^a^	√^a^	X	X	X	(√)^e^	X	(√)^e^
FcRn‐I	√^c^	√	√^b^, ^c^	√^b^	X	(√)^e^	X^d^	(√)^e^

*Note:* √ approved/recommended, X not approved/not recommended and (√) may be considered.

Abbreviations: APR: approved; GGR: German guideline recommendation for (highly) active gMG +/− thymectomy.

^a^ Ravulizumab and zilucoplan are approved for AChR‐Ab‐positive gMG. Eculizumab is approved only for treating refractory AChR‐Ab‐positive gMG in adults and children aged ≥ 6 years. As there is no universally accepted definition of ‘refractory’ gMG, the German guideline has deviated from this recommendation. Therefore, eculizumab has been included in the category of (highly) active gMG. Currently, eculizumab is the only option approved for use in children < 12 years.

^b^ Rozanolixizumab and nipocalimab, but not efgartigimod, are approved by EMA for MuSK‐Ab‐positive gMG.

^c^ Nipocalimab is the only FcRn‐I also approved by EMA for use in adults and adolescents aged ≥ 12 years.

^d^ In Japan, efgartigimod and rozanolixizumab are also approved for seronegative gMG. In the US, efgartigimod has been recently approved for seronegative gMG.

^e^ According to the German guideline, seronegative and LRP4‐Ab‐positive MG are generally treated like AChR‐Ab‐positive MG, but the guideline does not provide any specific recommendations for C5‐I and FcRn‐I [15].

### 3.1 Disease‐Activity Guided Treatment

Assessment of disease activity has gained increasing relevance for therapeutic decision‐making in gMG. The German guideline defines disease activity based on the severity of clinical symptoms, disease duration and response to prior therapies, differentiating between mild/moderate and (highly) active disease, the latter including ‘refractory gMG’ [15, 23]. International approaches, however, vary considerably, and a globally accepted standard for assessing disease activity is still lacking [24, 25]. According to the German guideline, (highly) active gMG, including refractory MG, is defined by persistent functionally relevant symptoms and/or recurrent severe exacerbations or myasthenic crises despite adequate symptomatic and disease‐modifying treatment. This includes patients with recurrent severe exacerbations or myasthenic crises requiring rescue therapy, as well as those with persistent symptoms relevant to daily living despite adequate treatment.

Treatment response and non‐response are commonly assessed using the Myasthenia Gravis Activities of Daily Living (MG‐ADL) score and the Quantitative Myasthenia Gravis (QMG) score, with clinically meaningful improvement commonly defined as a ≥ 2‐point reduction in MG‐ADL and a ≥ 3‐point reduction in QMG [26, 27]. These thresholds predate the pivotal phase 3 trials and were later incorporated into trial designs [1, 2, 3, 4, 5, 6, 7]. In clinical practice, patient‐specific treatment goals beyond these scales, including the patient acceptable symptom state (PASS), may also be considered; serial assessments across multiple follow‐up visits are recommended to confirm sustained therapeutic response [28, 29]. Available targeted immunotherapy options for (highly) active gMG currently comprise C5‐I (eculizumab, ravulizumab, zilucoplan, Table 1), FcRn‐I (efgartigimod, rozanolixizumab, nipocalimab, Table 1), CD20 B‐cell‐depleting therapies, especially RTX and rescue therapies including plasma exchange (PLEX), immunoadsorption (IA), IVIg and in certain cases high‐dose corticosteroids [24, 25]. Nipocalimab and inebilizumab are included to reflect recent regulatory developments but were not part of the expert consensus process due to pending EMA approval at the time of the expert meetings.

### 3.2 Consideration and Monitoring of Safety Parameters in Immunotherapy

Drug‐specific safety parameters must be assessed before initiation and at every switch of immunotherapy. These include monitoring of IgG levels under FcRn‐I and RTX therapy (especially when used in combination). Routine IgA testing prior to first administration of IVIg is not generally required but may be considered in selected patients with suspected severe IgA deficiency or a history of hypersensitivity reactions to blood products [30, 31].

As a general principle, patients should receive vaccinations according to national or local recommendations, ideally before initiation of immunotherapy whenever feasible. Vaccinations should preferably be administered 2–4 weeks before treatment initiation, with an interval of at least 2 weeks between vaccination and the start of therapy. For patients receiving complement inhibitors, particular attention should be paid to meningococcal vaccination (see below), as well as other recommended vaccinations against encapsulated bacteria, including *Haemophilus influenzae* type b and *Streptococcus pneumoniae* . If immediate initiation of complement inhibitor therapy is clinically required, the vaccination interval may be bridged with appropriate antibiotic prophylaxis. For FcRn‐I, data on vaccine responses remain limited; recommended vaccinations should therefore be completed before treatment initiation whenever feasible. In line with product‐specific prescribing information, live or live‐attenuated vaccines are generally not recommended during immunosuppressive treatment. In patients scheduled to receive B‐cell‐depleting therapy (e.g., rituximab or inebilizumab), vaccination status should be reviewed before treatment initiation, as vaccine responses may be reduced during and after B‐cell depletion. Whenever feasible, indicated vaccinations should be completed before treatment initiation; otherwise, vaccination should be postponed until B‐cell recovery. Live vaccines should generally be avoided until immune reconstitution has occurred (more details on meningococcal vaccination see chapters below and Supporting Information) [15, 16, 17, 18, 19, 20, 32, 33].

Baseline laboratory investigations for all immunotherapies should include HIV and hepatitis serology, as well as tuberculosis screening where indicated [15].

### 3.3 Initiating Add‐On Therapy With C5 and FcRn Inhibitors

#### 3.3.1 Role of C5 and FcRn Inhibitors in gMG

In Germany, C5‐I and FcRn‐I are used as add‐on therapies to standard treatment for (highly) active gMG in accordance with the national guideline and regulatory approvals (Table 1) [15]. Standard therapy typically comprises a combination of acetylcholinesterase inhibitors (AChEI), corticosteroids and steroid‐sparing immunosuppressive agents, such as azathioprine (AZA) or mycophenolate mofetil (MMF) [15]. Thymectomy represents a surgical option with class‐I‐evidence for patients with AChR‐Ab‐positive gMG aged 18–65 and a disease duration of no more than 5 years [34]. As there is no universally accepted definition of *standard therapy*, international practice varies considerably. For instance, Scandinavian guidelines recommend AZA and RTX as individual first‐line immunosuppressive therapies, whereas Japanese guidelines include tacrolimus [24, 25].

#### 3.3.2 Choosing an Appropriate Add‐On Therapy

**FIGURE 1:**
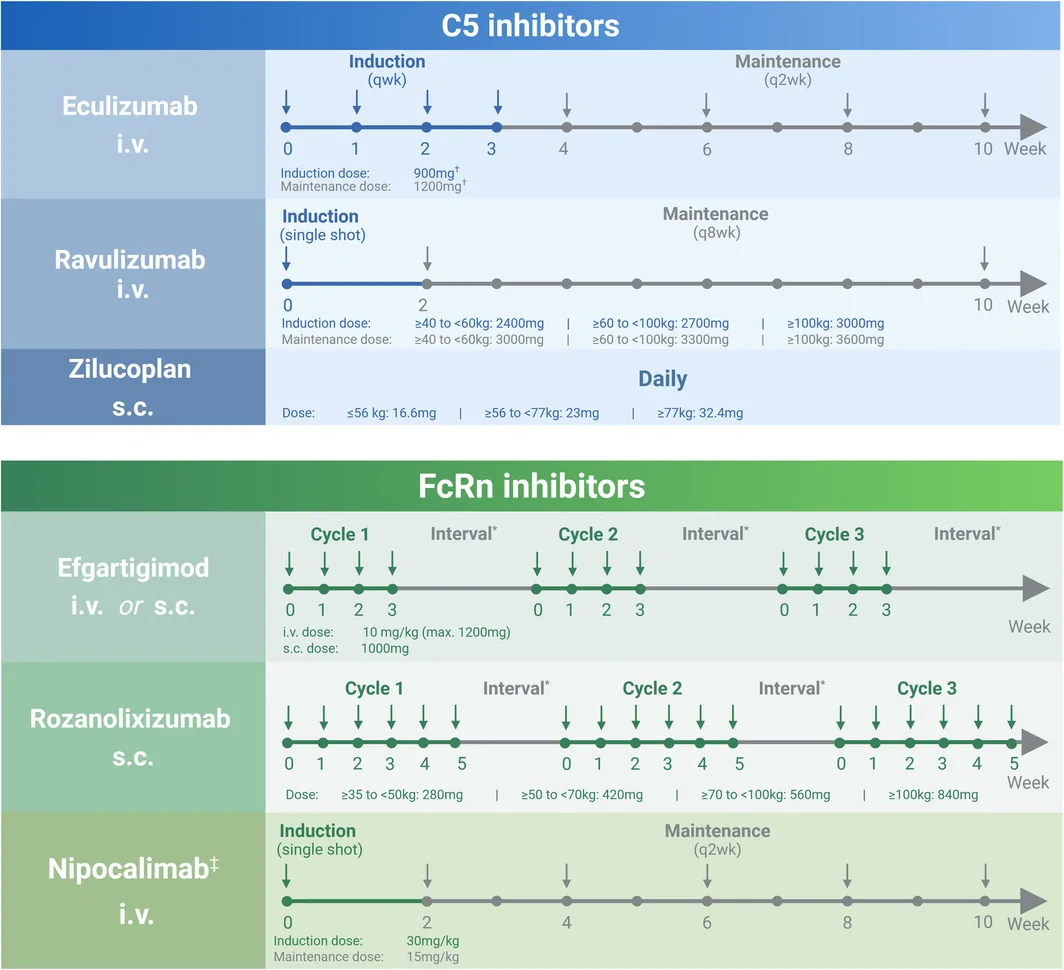
Application and dosage of the targeted immunotherapies C5‐I and FcRn‐I [6, 15, 16, 17, 18, 19, 20]. Patients should be advised that home infusion of eculizumab, ravulizumab, nipocalimab and efgartigimod i.v. may be considered for those who have tolerated hospital infusions well. In this case, good documentation of the treatment response must be ensured using the relevant scores [Myasthenia Gravis Activities of Daily Life (MG‐ADL), Quantitative Myasthenia Gravis (QMG), Myasthenia Gravis Quality of Life 15 revised (MG‐QoL15r)] [15, 17, 18, 19, 21]. Zilucoplan and rozanolixizumab injections/s.c.‐infusions can be self‐administered by the patient and/or another person with adequate training in s.c.‐injection administration [16, 20]. * Subsequent treatment cycles should be administered based on clinical assessment † In children and adolescents weighing less than 40 kg, the dose must be adjusted accordingly. ‡ Nipocalimab is approved for use in adults and adolescents aged ≥ 12 years with AChR‐Ab‐ or MuSK‐Ab‐positive gMG. Created in BioRender. Doksani, P. (2025) https://BioRender.com/cqpigeq.

The mode and frequency of administration of C5‐I and FcRn‐I are shown in Figure 1 [6, 15, 16, 17, 18, 19, 20]. Therapy selection should be based on a shared decision‐making process between physician and patient, considering individual disease characteristics, comorbidities and treatment logistics. Treatment should ideally be conducted at, or in close cooperation with, a specialised neuromuscular centre.

**FIGURE 2:**
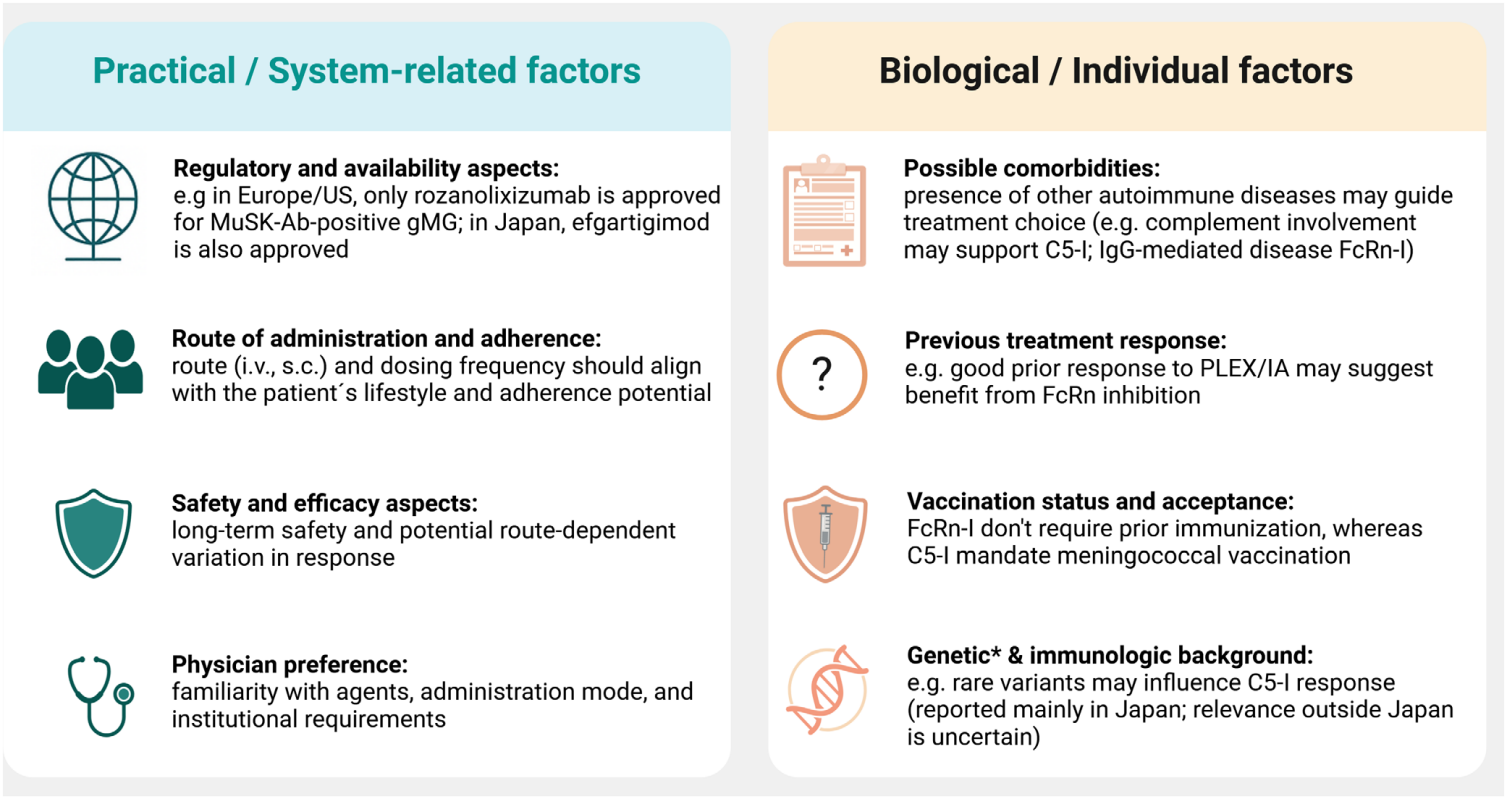
Decision criteria guiding the choice between C5‐I and FcRn‐I [35, 36, 37]. * The most relevant variant is C5 c.2654G>A (p.Arg885His), first described by Nishimura et al., with an allele frequency of approximately 3.5% in the Japanese population. Routine pre‐treatment genetic screening is currently not standard in clinical practice. Created in BioRender. Doksani, P. (2026) https://BioRender.com/3k3c2pd.

In clinical practice, several general criteria (e.g., regulatory framework, patient's lifestyle and adherence, safety and efficacy, or physician's experience) as well as other, less well‐established factors may also influence therapy selection in individual cases and may further guide the choice of add‐on therapy (Figure 2) [35].

### 3.4 Initiating Add‐On Therapy With C5 Inhibitors

#### 3.4.1 Use of Eculizumab, Ravulizumab and Zilucoplan in gMG

C5‐I can be considered for patients with (highly) active gMG in whom the disease pathophysiology is complement‐mediated, typically those who are AChR‐Ab‐positive [15, 17, 18, 20, 38]. Regulatory indications differ between regions. In the EU, eculizumab is approved specifically for refractory AChR‐Ab‐positive gMG, whereas ravulizumab and zilucoplan are approved as add‐on therapies without an explicit restriction to refractory disease [17, 18, 20]. In the United States, currently approved C5 inhibitors for gMG are not restricted to refractory disease by label, whereas in Japan approval may require an inadequate response to corticosteroids or non‐steroidal immunosuppressive therapies.

A clinical response is generally observed within the first 4 weeks after treatment initiation. In patients who do not achieve clinically meaningful improvement within 12 weeks, the decision to continue treatment or switch to an alternative therapy should be individualised [15, 17, 18, 20], as long‐term observational data from non‐placebo‐controlled studies suggest that delayed responses may occur, particularly in therapy‐refractory gMG patients treated with eculizumab or ravulizumab for several months or longer [39, 40].

In selected patients with LRP4‐Ab‐positive or seronegative gMG and evidence of complement‐mediated pathology (e.g., complement deposition at the neuromuscular junction) [38, 41], complement inhibition may be considered on an individual basis. This approach is supported by mechanistic evidence and is consistent with the current German MG guideline [15], which considers complement inhibition biologically plausible in LRP4‐Ab‐positive and seronegative gMG despite the lack of regulatory approval. However, clinical evidence remains limited to small case series and case reports [42, 43].

**Expert Consensus**

Recommendation (consensus): Add‐on therapy with C5‐I should be considered for patients with (highly) active gMG (including therapy‐refractory disease) who are positive for AChR autoantibodies. C5‐I should be taken into consideration in patients with (highly) active gMG (including therapy‐refractory disease) with LRP4‐antibody‐positive or seronegative status, provided complement‐mediated pathology has been demonstrated by complement deposition at the neuromuscular junction on muscle biopsy.

*Note*: The use of C5‐I in LRP4‐antibody‐positive or seronegative gMG is off‐label.

Recommendation (consensus): The use of C5‐I as monotherapy should be considered only in exceptional cases, for example, when standard immunotherapies are ineffective, associated with intolerable AEs, or contraindicated, including in the context of chronic infection or malignancy.

Recommendation (strong consensus): Treatment response should be assessed after 12 weeks of C5‐I therapy, as clinical response or non‐response can usually be determined by this time and used to guide further treatment planning.

*Note*: A non‐responder is a patient who fails to achieve clinically meaningful improvement despite adequate therapy (for details see above: Disease‐activity guided treatment).

#### 3.4.2 Safety Considerations With C5 Inhibitors

Meningococcal vaccination is mandatory prior to any C5‐I therapy [17, 18, 20]. Vaccination practices should comply with local guidelines, as these can vary greatly (e.g., concerning vaccination intervals). Treatment may begin no sooner than 2 weeks after vaccination against serogroups ACWY and after the first dose of vaccination against serogroup B. Patients who start C5‐I treatment less than 2 weeks after receiving a meningococcal vaccination must receive appropriate prophylactic antibiotics until 2 weeks after vaccination has been completed. Booster doses (usually after 3 years) should be administered as scheduled but should not delay ongoing treatment [15, 17, 18, 20, 33, 44]. Meningococcal vaccination significantly reduces the risk of infection, but does not eliminate it. Patients must therefore be informed about the residual risk of meningococcal disease, which most often manifests as fulminant sepsis [15, 33, 44]. A patient safety card must always be issued, when any C5‐I is prescribed [17, 18, 20]. For additional information on antibiotic prophylaxis and standby therapy, the reader is referred to Berthele et al. [44].

Pneumococcal vaccination is recommended for all patients receiving immunosuppressive therapy, particularly those aged over 60 years [33].

**Expert Consensus**

Recommendation (consensus): Prior to initiating C5‐I therapy, patients must be thoroughly informed about the clinical signs of meningococcal infection and the immediate actions required. Although rare, severe meningococcal infections may develop within hours despite prior vaccination. Patients who are able may be advised to initiate standby antibiotic therapy at home. In all cases, rapid access to emergency medical care must be ensured.

Pancreatic enzyme elevations have been reported during treatment with zilucoplan. Baseline assessment of serum lipase and, if clinically indicated, amylase may therefore be considered before treatment initiation, particularly in patients with a history of pancreatic disease. Follow‐up monitoring should be guided by clinical symptoms and individual risk factors. As clinical experience accumulates, the need for routine pancreatic enzyme monitoring should be further evaluated.

### 3.5 Initiating Add‐On Therapy With FcRn Inhibitors

#### 3.5.1 Use of Efgartigimod and Rozanolixizumab in gMG

Efgartigimod is currently approved by EMA for the treatment of AChR‐Ab‐positive gMG, while rozanolixizumab and nipocalimab are approved for AChR‐Ab‐positive and MuSK‐Ab‐positive gMG. From a pathophysiological perspective, FcRn‐I may be considered in (highly) active gMG irrespective of antibody status, as their mode of action reduces circulating pathogenic IgG autoantibodies regardless of the specific target antigen [45, 46]. A dedicated phase 3 study investigating efgartigimod in AChR‐Ab‐negative gMG has recently been completed, but peer‐reviewed data are not yet available [22].

**Expert Consensus**

Recommendation (strong consensus): Add‐on therapy with FcRn‐I should be considered for patients with (highly) active gMG (including therapy‐refractory disease) who are positive for AChR‐ and MuSK‐autoantibodies. FcRn‐I should be taken into consideration in patients with (highly) active gMG (including therapy‐refractory disease) with LRP4‐antibody‐positive or seronegative status.

*Note*: The use of FcRn‐I in LRP4‐antibody‐positive and seronegative patients and the use of efgartigimod in MuSK‐antibody‐positive patients is off label. Nipocalimab is approved for use in adults and adolescents aged ≥ 12 years with AChR‐Ab‐ or MuSK‐Ab‐positive gMG.

Efgartigimod and rozanolixizumab are given in individualised treatment cycles, while nipocalimab is given biweekly in a fixed schedule. In the pivotal ADAPT trial, efgartigimod was administered in 4‐week treatment cycles, with re‐treatment permitted only in the event of clinical deterioration and no earlier than 7 weeks after the first infusion of the preceding cycle. This regimen was adopted in the prescribing information, which does not stipulate fixed re‐initiation intervals [2]. For rozanolixizumab, only one single treatment cycle was evaluated in the registration study, and re‐treatment was performed upon clinical worsening as needed; accordingly, no defined minimum interval between cycles is specified in the label [5]. In the ADAPT trial, 36% of patients started a new efgartigimod cycle after < 6 weeks, 27% after 6–9 weeks and 37% after ≥ 9 weeks [2, 16, 19]. Real‐world data suggest that most patients require earlier retreatment, often within 6 weeks, occasionally even after fewer than 4 weeks [8, 47, 48]. Thus, subsequent treatment cycles should be administered based on clinical assessment, with intervals varying substantially between patients and potentially also within the same patient over time. Importantly, experts generally agree that treatment should aim to maintain a sustained clinical response rather than waiting for overt clinical deterioration.

**Expert Consensus**

Recommendation (strong consensus): Timing of a new FcRn‐I cycle should be individualised.

31% of experts favoured initiating a new treatment cycle immediately at the first signs of clinical worsening (no consensus), whereas 69% recommended scheduling the next cycle according to the previous treatment course and starting immediately before the anticipated onset of clinical deterioration (majority vote).

#### 3.5.2 Safety Considerations With FcRn Inhibitors

FcRn‐I transiently reduces serum IgG levels, which may increase the risk of infection. IgG reductions of up to 70% vs. baseline have been observed in clinical studies [16, 19]. However, absolute IgG levels are more clinically relevant than relative reductions, and long‐term data on treatment‐related transient hypogammaglobulinaemia remain limited, and no validated cut‐off values have been defined. Concomitant immunosuppression and infection history should be considered individually [49].

IgG levels should be assessed prior to treatment initiation. In the pivotal trials, minimum IgG levels of > 6 g/L for efgartigimod and > 5.5 g/L for rozanolixizumab were required for study participation. In clinical practice, these values may serve as general reference points rather than strict treatment thresholds. Lower IgG levels are not an absolute contraindication, and decisions regarding treatment initiation or continuation should be individualised, taking into account disease activity, infection risk and the degree of IgG reduction. As a practical consideration IgG levels > 5 g/L before treatment initiation and > 4 g/L before subsequent treatment cycles [16, 19] may serve as pragmatic reference values, although these values should not be regarded as evidence‐based cut‐offs. IgG monitoring is not mandatory but advised before starting a new treatment cycle, particularly in patients with frequent infections, low baseline IgG, prior B‐cell‐depleting therapy, or recent PLEX [49].

Meningococcal vaccination is not required but may be advisable to allow a rapid switch to C5‐I in case of insufficient response or relevant AEs [15, 16, 19, 33].

**Expert Consensus**

Recommendation (strong consensus): FcRn‐I therapy should be accompanied by monitoring of infections and IgG levels, particularly before initiation and, if needed, at the beginning and end of individual treatment cycles depending on the values obtained.

## 4 Switching Within and Between C5 and FcRn Inhibitors

### 4.1 General Principles

During ongoing treatment with C5‐I and FcRn‐I, both intra‐ and interclass switches may be considered for clinical or practical reasons. There is a lack of data from clinical trials and real‐world studies. Accordingly, the following statements are based on the experts' collective clinical experience.

Intraclass switching may be appropriate in patients who previously responded to the class‐specific mechanism of action but experience a *wearing‐off* or *end‐of‐dose* phenomenon before the end of the dosing interval, warranting transition to an agent with shorter administration intervals. Switching may also be considered for administration‐related reasons, for example, from subcutaneous (s.c.) to intravenous (i.v.) application in case of local skin reactions, or conversely, from i.v. to s.c. application to enhance patient autonomy and treatment flexibility.

Interclass switching may be appropriate when the prior treatment mode of action proves ineffective at the approved dosage and duration, if intolerable adverse effects occur, or when patient preferences regarding route or frequency of administration require reconsideration [35]. From the patient's perspective, a cyclical treatment regimen may be preferred over continuous administration, or vice versa. If a patient has another antibody‐mediated autoimmune disease that can be successfully managed with either C5‐I (e.g., neuromyelitis optica spectrum disorder) or FcRn‐I (e.g., chronic inflammatory demyelinating polyradiculoneuropathy), or if a pathophysiological rationale for cross‐efficacy exists, an interclass switch may be justified [50]. Before changing therapy, the standards and precautions outlined in the previous chapters should be carefully considered.

**Expert Consensus**

Recommendation (strong consensus): An intraclass switch should be considered in patients with a documented response to the class‐specific mode of action who develop a ‘wearing‐off’ or ‘end‐of‐dose’ phenomenon before the end of the dosing interval, warranting transition to a preparation with shorter intervals. An intraclass switch should also be considered for administration‐related reasons, such as switching to i.v. administration in the event of skin reactions during s.c. therapy or switching from i.v. to s.c. administration to increase patient autonomy.

Recommendation (consensus): An interclass switch should be considered if therapy is ineffective at the appropriate dosage and duration, if intolerable side effects occur, or for patient preferences regarding type and frequency of administration.

Recommendation (strong consensus): A switch to IVIg interval therapy should be considered in cases where targeted immunotherapies fail to provide adequate clinical efficacy.

*Note*: IVIg interval therapy is off label.

### 4.2 Intraclass Switching

#### 4.2.1 Switching Within the C5 Inhibitor Class

Switching within the C5‐I class is typically undertaken in patients with a sustained response to complement inhibition who require optimisation of dosing intervals or a different route of administration, rather than in cases of insufficient efficacy or class‐related AEs. No safety interval is required when switching within the C5‐I class, as the mechanism of action is shared. However, dosing differences between agents (e.g., between eculizumab and ravulizumab) must be considered. A short interval of a few days may be useful to distinguish AEs potentially related to either compound [35].

Clinically relevant reasons for intraclass switches include: [12, 35]

- Transition from long‐acting to short‐acting therapy (e.g., ravulizumab → eculizumab or zilucoplan) to improve controllability or manage end‐of‐dose effects
- Switching from i.v. to s.c. administration to enhance patient autonomy, or conversely in the case of local injection‐site reactions
- Adjusting dosing frequency or route of administration according to patient preference (e.g., low‐frequency i.v. versus high‐frequency s.c. dosing)

#### 4.2.2 Switching Within the FcRn Inhibitor Class

Intraclass switching within FcRn‐I may be considered in patients who have previously responded to this mode of action but develop local AEs or require a change in administration route or dosing frequency or wish to enhance their autonomy. From a mechanistic point of view, no safety interval is required when switching within this class.

Typical clinical scenarios include:

- Switching from efgartigimod i.v. to efgartigimod s.c. (or vice versa) to improve convenience or local tolerability
- Transition from efgartigimod to rozanolixizumab in patients with insufficient duration of effect
- Adjustment of dosing frequency to minimise fluctuations or end‐of‐dose effects

### 4.3 Interclass Switching

#### 4.3.1 Interclass Switching From C5 to FcRn Inhibitors

A switch from ongoing C5‐I therapy to FcRn‐I may be considered in patients who fail to achieve sufficient clinical response, in case of intolerable AEs, or following a serious infection with encapsulated bacteria (e.g., meningococcal or pneumococcal infection despite vaccination).

In patients not responding to C5‐I, FcRn‐I offers a mechanistically broader approach targeting additional antibody‐mediated pathways [51]. Given the distinct mechanisms of action and lack of overlapping pharmacodynamics, no washout period is required when switching between these classes. However, since concurrent reduction of complement activity and IgG levels could transiently increase infection risk, a short interval of 1–2 weeks may be considered to facilitate attribution of potential AEs to one agent.

#### 4.3.2 Interclass Switching From FcRn to C5 Inhibitors

A switch from FcRn‐I to C5‐I therapy may be appropriate for patients with AChR‐Ab‐positive gMG. Here, potential pharmacodynamic interactions must be considered: The inhibition of FcRn can accelerate the degradation of monoclonal antibodies such as eculizumab or ravulizumab [17, 19, 52]. Therefore, an interval of approximately 1–3 weeks is recommended before initiating monoclonal antibody‐based C5‐I therapy. No washout period is required when switching to the macrocyclic peptide C5‐I zilucoplan [35].

**Expert Consensus**

Recommendation (consensus): Omission of a washout period can be considered when switching between C5‐I and FcRn‐I, given the distinct mechanisms of action of these inhibitors and the absence of overlapping therapeutic effects.

## 5 Switching to Other Immunomodulatory Therapies and Rescue Procedures

In clinical practice, targeted inhibitors may need to be sequenced with other intensified immunomodulatory therapies or rescue procedures. In addition to C5‐I and FcRn‐I, B‐cell‐targeted therapy with RTX and interval immunoglobulin therapy (IVIg or SCIg) remain established options within the therapeutic continuum in gMG. These agents are typically used as intensified or adjunctive treatments in patients with incomplete response to or contraindications for standard or even targeted immunotherapies. RTX acts upstream of C5‐I and FcRn‐I within the autoimmune cascade, whereas IVIg and SCIg exert broad, pleiotropic immunomodulatory effects and serve primarily as rescue options [15, 24, 53].

The recommendations presented here focus on C5‐I and FcRn‐I but should be interpreted within the broader therapeutic framework that includes these intensified treatment options. Note: The use of RTX and SCIg is off‐label in gMG. Further details and recommendations regarding RTX and IVIg/SCIg are provided in the Supporting Information.

### 5.1 Switching From and to Rituximab

#### 5.1.1 Switching From C5 Inhibitors to Rituximab

RTX is not considered a standard treatment option when discontinuing C5‐I therapy but should rather be regarded as an alternative to conventional steroid‐sparing immunosuppressants such as AZA, MMF, methotrexate (MTX), or cyclosporine A (CSA). Combination therapy of C5‐I with RTX has been used in clinical practice. However, this approach has not yet been investigated in clinical studies due to possible interactions (including complement‐dependent cytotoxicity of RTX) [54]. The following considerations are therefore based on expert clinical experience. Because the primary focus of this paper is switching within and between C5‐I and FcRn‐I, more detailed considerations regarding RTX and combined RTX/C5‐I therapy are provided in the Supporting Information.

#### 5.1.2 Switching From FcRn Inhibitors to Rituximab

FcRn‐I and RTX may interact pharmacodynamically. FcRn inhibition can accelerate the clearance of monoclonal antibodies such as RTX, potentially reducing therapeutic efficacy. Moreover, the combination of B‐cell depletion and reduced IgG levels may increase the risk of hypogammaglobulinaemia and subsequent infections [55, 56, 57, 58]. Therefore, when switching from FcRn‐I to RTX, allowing a sufficient time interval for serum IgG levels to normalise (preferably > 5 g/L) is important [56, 59, 60].

RTX is primarily considered an alternative to conventional steroid‐sparing immunosuppressants rather than to FcRn‐I therapy. However, RTX has demonstrated robust clinical efficacy in MuSK‐Ab‐positive gMG across multiple observational studies and real‐world cohorts (Supporting Information). This efficacy is attributable to RTX's ability to deplete B cells and short‐lived plasmablasts responsible for producing pathogenic IgG4 autoantibodies in MuSK‐Ab‐positive gMG. Therefore, in MuSK‐Ab‐positive patients who fail to achieve adequate benefit from FcRn inhibition, switching to RTX is justified [61].

**Expert Consensus**

Recommendation (strong consensus): A switch from FcRn‐I to RTX must be considered in patients with MuSK‐Ab‐positive gMG who fail to achieve an adequate response.

*Note*: The use of RTX and efgartigimod for this indication is off label.

#### 5.1.3 Switch From Rituximab or Combining With C5 or FcRn Inhibitors

Switching from RTX to C5‐I or FcRn‐I is rarely required in clinical practice. More commonly, C5‐I or FcRn‐I are used as add‐on therapies to RTX, like their combination with other steroid‐sparing immunotherapies (AZA, MMF, CSA and MTX). However, evidence from controlled studies is limited, as RTX medication was excluded from pivotal trial designs. Therefore, the following statements reflect the panel's collective clinical experience.

C5‐I may be preferable when a predominantly complement‐mediated mechanism is indicated (e.g., AChR‐Ab‐positive gMG) or when IgG levels are already low after RTX treatment, as FcRn‐I might further reduce IgG concentrations. Conversely, FcRn‐I may be favoured when disease activity appears driven by circulating IgG antibodies without clear complement involvement (e.g., MuSK‐Ab‐positive gMG), or when vaccination status or infection history contraindicates complement inhibition. Before combining or sequencing these agents, potential additive risks such as hypogammaglobulinaemia and subsequent infections should be carefully evaluated and treatment timing adjusted accordingly. When initiating FcRn‐I after RTX, IgG levels should exceed 5 g/L. Further information on use and safety considerations of RTX in gMG can be found in the Supporting Information.

**Expert Consensus**

Recommendation (consensus): MuSK‐Ab‐positive gMG patients should only be switched from RTX to an FcRn‐I if RTX proves insufficiently effective or if side effects are unacceptable.

*Note*: The use of RTX and efgartigimod for this indication is off label.

Recommendation (strong consensus): IgG serum levels should be measured before initiating FcRn‐I therapy, as levels may be reduced by prior RTX treatment. If IgG levels are below the recommended threshold at the start of a treatment cycle, FcRn‐I therapy should not be administered.

### 5.2 Switching From and to IVIg/SCIg

#### 5.2.1 Switching From Interval IVIg/SCIg to C5 or FcRn Inhibitors

In AChR‐Ab‐positive gMG, a switch from interval IVIg/SCIg to FcRn‐I or C5‐I is evidence‐based and in line with current approvals [16, 17, 18, 19, 20]. In MuSK‐Ab‐positive gMG, switching from interval IVIg/SCIg to FcRn‐I may be considered [15]. Importantly, potential pharmacodynamic interactions between IVIg/SCIg and FcRn‐I should be considered, since IVIg competes for FcRn binding [62] and may transiently affect the pharmacokinetics and efficacy of FcRn‐targeting agents. However, this mechanism may also have a beneficial bridging effect, potentially reducing disease activity and infection risk during therapy transition (see also ‘Effects of rescue therapy on FcRn inhibitors’) [63].

When switching from interval IVIg/SCIg therapy to C5‐I or FcRn‐I, an interval should be maintained between the last immunoglobulin administration and the start of targeted immunotherapies. An interval of one to 4 weeks can serve as a practical guideline.

The use of IVIg or SCIg for interval therapy is off label for gMG. Further information on interval IVIg/SCIg therapy in gMG can be found in the Supporting Information.

**Expert Consensus**

Recommendation (strong consensus): Patients who are clinically stable on IVIg or SCIg interval therapy without intolerable side effects should not be switched to C5‐I or FcRn‐I.

*Note*: The use of IVIg or SCIg interval therapy is off‐label in gMG.

Recommendation (strong consensus): In patients with AChR‐Ab‐positive, highly active gMG who do not achieve minimal disease activity with IVIg or SCIg interval therapy, a switch to a C5‐I or an FcRn‐I should be considered.

## 6 Rescue Therapies

### 6.1 Switching From Rescue Therapy (PLEX/IA/IVIG) to C5 or FcRn Inhibitors

Early initiation of C5‐I or FcRn‐I may be beneficial in patients experiencing severe exacerbations or myasthenic crisis who respond inadequately to conventional rescue therapies (PLEX, IA, or IVIg). In the pivotal trials supporting the approval of all currently authorised active substances, patients experiencing myasthenic crisis (Myasthenia Gravis Foundation of America [MGFA] Score V) were excluded [1, 2, 3, 4, 5, 6]. Consequently, there is a substantial lack of high‐quality evidence regarding the efficacy and safety of these therapies in this high‐risk population. As a result, treatment recommendations for patients in myasthenic crisis necessarily rely on expert consensus and real‐world clinical experience. Within this framework, targeted immunotherapies are considered promising options for stabilising patients during or shortly after crises, given that both terminal complement inhibition and IgG depletion act rapidly and are supported by encouraging case reports [64, 65, 66, 67, 68, 69].

When initiating FcRn‐I therapy following apheresis, serum IgG levels should be carefully monitored (see respective safety considerations). Following rescue IVIg treatment, potential pharmacodynamic interactions with FcRn‐I should be considered, as the transient increase in circulating IgG may affect FcRn‐mediated recycling and the kinetics of IgG reduction. However, the clinical relevance of this interaction remains uncertain. Suggested intervals for initiating or continuing FcRn‐I therapy after PLEX, IA, or IVIg administration are outlined below:

**Expert Consensus**

Recommendation (strong consensus): Early use of C5‐I or FcRn‐I should be considered in the management of severe exacerbations and myasthenic crisis if there is an inadequate response to exacerbation therapy (PLEX/IA/IVIg).

### 6.2 Rescue Therapy Management During Treatment With C5 or FcRn Inhibitors

Phase‐3 trials of FcRn‐I and C5‐I demonstrated a significant reduction in the frequency of severe exacerbations and myasthenic crises, although these events were not completely eliminated [1, 2, 3, 4, 5]. Consequently, occurrence of a severe exacerbation or myasthenic crisis does not in itself warrant a change in therapy. In patients who experience recurrent exacerbations or MG crises despite ongoing treatment with C5‐I or FcRn‐I, switching between therapeutic classes (C5‐I ↔ FcRn‐I) may be considered following a thorough evaluation of potential precipitating factors. This decision should be guided by prior therapeutic response, whether precipitating factors can be identified and addressed, as well as cumulative burden, frequency and severity of exacerbations [35].

**Expert Consensus**

Recommendation (consensus): In patients who experience recurrent exacerbations of myasthenic symptoms or myasthenic crises under C5‐I or FcRn‐I therapy, requiring rescue treatments (e.g., IVIg, PLEX, IA), a switch between therapeutic classes—e.g., from C5‐I to FcRn‐I or vice versa—should be considered after a thorough evaluation of potential triggers.

### 6.3 Effect of Rescue Therapy on C5 Inhibitors

During PLEX or IA, eculizumab and ravulizumab as monoclonal antibodies are removed from the patient's plasma, while IVIg may reduce their circulating levels, necessitating administration of an additional dose. In contrast, concentrations of the macrocyclic peptide zilucoplan remain unaffected [70]. In routine clinical practice, during a course of PLEX or IA, C5‐I redosing is generally not performed after each individual session as outlined in the prescribing information; rather, the full induction regimen is reinitiated following completion of the apheresis series. When continuation of C5‐I therapy is planned following rescue therapy, the standard induction regimen can be reinitiated as soon as possible [17, 18, 20].

**Expert Consensus**

Recommendation (consensus): When continuation of C5‐I therapy with eculizumab or ravulizumab is planned after completion of rescue treatment, the standard induction regimen should be reinitiated. Restart is advised as soon as possible (recommended time frame after IVIg/PLEX/IA: 1‐4 weeks). Treatment with zilucoplan may be continued during rescue therapy.

### 6.4 Effect of Rescue Therapy on FcRn Inhibitors

If an exacerbation occurs during FcRn‐I therapy, IVIg may be administered as rescue therapy to stabilise disease activity, prevent excessive IgG depletion and avoid the need for further FcRn‐I dose adjustments. In case of an impending or manifest myasthenic crisis, PLEX or IA are preferred rescue therapies, with close monitoring of IgG levels being essential. IVIg substitution may be necessary.

Since PLEX, IA and IVIg may reduce circulating FcRn‐I concentrations and may therefore diminish therapeutic efficacy, follow‐up therapy should be scheduled promptly after completion of rescue intervention. A new treatment cycle can be initiated once the patient is clinically stable and IgG levels have adequately recovered. In cases of severe hypogammaglobulinaemia (e.g., < 4 g/L), particularly when accompanied by infectious complications following PLEX or IA, IVIg substitution may be indicated to restore immunoglobulin levels before reinitiating FcRn‐I treatment [16, 19].

**Expert Consensus**

Recommendation (consensus): Continuation of FcRn‐I therapy immediately after rescue treatment should be considered (recommended time frame after PLEX/IA/IVIg: 1–4 weeks). IgG levels should be assessed before initiating therapy to avoid severe hypogammaglobulinaemia.

## 7 De‐Escalation of Treatment With C5 or FcRn Inhibitors

The ultimate therapeutic goal in the management of gMG is a complete and stable remission. This is defined as sustained absence of symptoms without the need for ongoing immunotherapy. In clinical practice, however, the primary goal is to induce pharmacological remission or achieve minimal manifestation status while reducing the corticosteroid dose to low‐dose (≤ 7.5 mg prednisolone equivalent per day) or, if possible, discontinuing steroid therapy in the long term. Under C5‐I and FcRn‐I treatment, corticosteroid doses can often be reduced and, in some cases, discontinued, which represents a reasonable therapeutic goal given the well‐known adverse effect profile of corticosteroids [8, 71].

Once disease stability has been achieved, the next clinical question is whether, and how, immunomodulatory therapy can be safely tapered or discontinued. As systematic evidence on de‐escalation strategies is currently lacking, clinical judgement based on individual disease characteristics remains crucial. In patients receiving both targeted therapy and conventional immunosuppression, de‐escalation is typically pursued first by reducing the intensity of the targeted therapy, while maintaining the backbone therapy. For both C5‐I and FcRn‐I, gradual extension of treatment intervals is generally preferred over abrupt discontinuation, particularly in patients with long‐standing disease or a history of relapse following treatment interruption. Conventional immunosuppressive therapies such as AZA or MMF should typically be maintained or tapered slowly according to individual response and tolerability, as their continuation may facilitate subsequent reduction of C5‐I or FcRn‐I. De‐escalation should always be guided by regular clinical monitoring to detect early signs of recurrence. If disease activity increases, reinitiation of the previously effective regimen should be considered to regain disease control.

Given the potential long‐term risks, including malignancy, de‐escalation of concomitant steroid‐sparing immunosuppressive therapies (e.g., AZA) may be considered in selected patients, even if continued treatment with an FcRn‐I or C5‐I remains necessary. In such cases, any dose reduction should be implemented cautiously and in a gradual, stepwise manner.

**Expert Consensus**

Recommendation (majority vote by 75%): Standard immunosuppressive therapies such as AZA and MMF should be continued or tapered only gradually according to individual response and tolerability. Continuation of these agents may facilitate subsequent de‐escalation of C5‐I, FcRn‐I, or interval IVIg/SCIg therapy during long‐term management.

## 8 Management of Juvenile MG (jMG)

### 8.1 Treatment Options and Ongoing Studies for (Highly) Active jMG

For general management of jMG, reference is made to the results of the 242nd ENMC International Workshop on Diagnosis and Treatment of jMG as well as the German guideline, which provide detailed recommendations on diagnostic and therapeutic approaches [15, 72]. The range of approved therapies for (highly) active jMG remains limited compared with adults. Currently, eculizumab is approved for patients aged ≥ 6 years with AChR‐Ab‐positive refractory gMG [15, 17, 73]. Nipocalimab is approved for adolescents aged 12 years and older with AChR‐Ab‐ or MuSK‐Ab‐positive gMG [21].

The use of the following C5‐I and FcRn‐I, which are currently used in adults, is being investigated in children and adolescents with jMG in ongoing clinical trials: ravulizumab [74, 75], zilucoplan [76], efgartigimod [77, 78, 79], rozanolixizumab [80].

### 8.2 Transition From Paediatric to Adult Care

The transition of jMG patients should follow a structured, interdisciplinary approach starting around age 16 and tailored to disease complexity. Transition clinics and structured programs such as the Essen Transition Model have shown good feasibility [81, 82]. Attention should be given to prior steroid use, as negative perceptions in childhood may affect future adherence. Escalation and response to immunosuppressive therapy should be carefully documented to guide later de‐escalation. In the absence of specific evidence‐based recommendations, treatment during transition should align with adult gMG management principles.

**Expert Consensus**

Recommendation (consensus): Medications (e.g., C5‐I, FcRn‐I) used in adults with (highly) active gMG should be considered for patients with (highly) active jMG, including those with therapy‐refractory disease courses.

*Note*: Eculizumab is approved for children aged 6 years and older with AChR‐Ab‐positive, refractory gMG. Nipocalimab is approved for use in adolescents aged 12 years and older with AChR‐Ab‐ or MuSK‐Ab‐positive gMG. The use of other C5‐I and FcRn‐I for this indication is off label.

## 9 Conclusion and Limitations

The recommendations for switching therapy were primarily developed for patients receiving FcRn‐I and C5‐I in Germany and Austria. The majority of underlying clinical principles are likely transferable to other healthcare settings. However, regulatory approval, reimbursement frameworks and national guidelines vary across countries. Implementation should therefore always be aligned with local regulations and clinical guidelines. This is particularly important as several of the therapies considered here are used off‐label even within Germany and Austria.

A key strength of these recommendations is their holistic approach, encompassing not only modern targeted immunotherapies but also established standard treatments, including relevant rescue therapies (also encompassing off‐label options). In addition, the explicit inclusion of paediatric patients addresses an important gap in clinical guidance and supports more comprehensive, real‐world decision‐making across age groups.

However, several limitations must be acknowledged. For many of the recommendations, high‐quality evidence remains limited, and expert consensus was therefore a major guiding factor in their development. In addition, these recommendations do not reflect clinical experience with the recently approved therapies for generalised myasthenia gravis, nipocalimab, and inebilizumab and their role was therefore only considered to a limited extent.

Consequently, neither sufficient evidence nor practical experience within the expert panel was available for these agents in the context addressed here.

In light of these considerations, the recommendations should be interpreted in the context of the available evidence and reviewed regularly to incorporate emerging data and newly available therapeutic options, ensuring their continued relevance and clinical utility.

## Author Contributions

**Andreas Meisel:** writing – review and editing, conceptualization, funding acquisition, writing – original draft, methodology, supervision, project administration, investigation, visualization. **Adela Della Marina:** writing – review and editing, methodology, conceptualization, investigation. **Paolo Doksani:** writing – review and editing, visualization, investigation. **Tim Hagenacker:** writing – review and editing, methodology, conceptualization, investigation. **Christoph Kleinschnitz:** writing – review and editing, methodology, conceptualization, investigation. **Heidrun H. Krämer:** writing – review and editing, methodology, conceptualization, investigation. **Jan D. Lünemann:** writing – review and editing, methodology, conceptualization, investigation. **Sven G. Meuth:** writing – review and editing, methodology, conceptualization, investigation. **Tobias Ruck:** writing – review and editing, methodology, conceptualization, investigation. **Ulrike Schara‐Schmidt:** writing – review and editing, methodology, conceptualization, investigation. **Christiane Schneider‐Gold:** writing – review and editing, methodology, conceptualization, investigation. **Benedikt Schoser:** writing – review and editing, methodology, conceptualization, investigation. **Michael Schroeter:** writing – review and editing, methodology, conceptualization, investigation. **Charlotte Schubert:** writing – review and editing, methodology, conceptualization, investigation. **Jörn Peter Sieb:** writing – review and editing, methodology, conceptualization, investigation. **Fritz Zimprich:** writing – review and editing, methodology, conceptualization, investigation. **Jana Zschüntzsch:** writing – review and editing, methodology, conceptualization, investigation. **Sarah Hoffmann:** writing – review and editing, conceptualization, funding acquisition, writing – original draft, methodology, supervision, project administration, visualization, investigation.

## Funding

This work was supported by Alexion Pharma Germany GmbH, which funded medical writing and editorial assistance provided by dpmed GmbH; the sponsor had no influence on the content.

## Ethics Statement

This work is based on previously conducted studies and does not contain any new studies with human participants or animals performed by any of the authors.

## Conflicts of Interest

A.M.: speaker, consultancy honoraria or financial research support from Alexion Pharmaceuticals, Argenx, Amgen, Axunio, Desitin, Grifols, Janssen, Merck, Novartis, Octapharma, Sanofi and UCB. A.D.M.: speaker's honoraria from Alexion. P.D.: speaker, consultancy honoraria or financial research from argenx, alexion and UCB. T.H.: speaker, consultancy honoraria or financial research support Alexion, Amgen, argenx, Astra Zeneca, Bayer, Biogen, Janssen/Johnson‐Johnson, Novartis, Roche and Sanofi‐Aventis. C.K.: speaker, consultancy honoraria or financial research support from Alexion, Amgen, argenx, Astra Zeneca, Bayer, Biogen, Bristol Myers‐Squibb, C.T.I., Daiichi Sankyo, Esai, GE Healthcare, Horizon Therapeutics, Hexal, Janssen/Johnson‐Johnson, Lilly, Merck, Neuroaxpharm, Novartis, Pfizer, Roche, Sanofi‐Aventis, Sandoz, Stada, Teva and Viatris. H.H.K.: speaker, consultancy honoraria or financial research support from Alexion, Sanofi, Grifols, Roche, argenx, UCB and Amgen. J.D.L.: speaker, consultancy honoraria or financial research support from Abbvie, Alexion, Adivo, Amgen, argenx, Biogen, CSL Behring, Grifols, Janssen‐Cilag, Merck, Moderna, Novartis, Octapharma, Roche, Sanofi, Takeda and UCB Pharma. S.G.M.: speaker, consultancy honoraria or financial research support from argenx, Alexion, Almirall, Amicus Therapeutics Germany, Bayer Health Care, Biogen, BioNtech, BMS, Celgene, Demecan, Desitin, Diamed, Diaplan, DIU Dresden, Fresenius Medical Care, Genzyme, Hexal AG, HERZ Burgdorf, IGES, Impulze, Janssen Cilag, KW Medipoint, MedDay, Merck, MICE, Mylan, Neuraxpharm, Neuropoint, Novartis, Novo Nordisk, ONO Pharma, Oxford PharmaGenesis, QuintilesIMS, Roche, Sanofi‐Aventis, STADA, Chugai Pharma, Teva, UCB, Viatris and Xcenda. T.R.: speaker, consultancy honoraria or financial research support from Amgen, argenx, Alexion, J&J, Merck, Novartis, Roche, UCB, Sanofi and SERB. U.S.‐S.: speaker or consultancy honoraria from Alexion. C.S.‐G.: speaker's or consultant's honoraria from Amicus Therapeutics, argenx, Alexion, Amgen, Hormosan Pharma, Janssen/Johnson&Johnson, Kyverna, Lupin Pharmaceuticals, Novartis, Sanofi‐Genzyme, Roche and UCB. B.S.: speaker or consultancy honoraria or financial research support from alexion, argenx, Astellas, Avidity, AMDA Foundation, Amicus Therapeutics Inc., Dyne, J&J, Roche and Sanofi. M.S.: speaker's honoraria from Alexion, argenx, Advio, Biogen, Grifols, Novartis, Roche, Sanofi and UCB. C.S.: speaker or consultancy honoraria Alexion, argenx and Johnson&Johnson. J.P.S.: speaker's or consulting honoraria from Alexion, argenx, Axunio, Hormosan, Janssen‐Cilag, Roche, SERB, Temmler and UCB. F.Z.: speaker's honoraria from argenx, UCB, Alexion, CSL‐Behring. J.Z.: speaker, consultancy honoraria or financial research support from Alexion, argenx, Amicus, CSL Behring, J&J, Kedrion, Sanofi and UCB. S.H.: speaker, consultancy honoraria or financial research support from Alexion, argenx, UCB, Grifols, Kyverna, Merck, Novartis, Roche and J&J.

## Supporting information


**Data S1:** ene70747‐sup‐0001‐Supinfo1.docx.
**Chapter 1:** Initiation of rituximab treatment in generalised myasthenia gravis.
**Chapter 2:** Role of intravenous and subcutaneous immunoglobulins in generalised myasthenia gravis.

## Data Availability

Data sharing not applicable to this article as no datasets were generated or analysed during the current study.
