# Supplementary material for: Practical Guidance on Initiating and Switching Targeted Immunotherapies in Generalised Myasthenia Gravis: A German–Austrian Expert Opinion Paper
Source: Eur J Neurol. 2026 Sep 9;33(9):e70747. doi: 10.1111/ene.70747 (PMC13559022; doi:10.1111/ene.70747)
Supplement: Supplementary file 1 [file ENE-33-e70747-s001.docx]

# **Supporting Information**

## **Chapter 1: Initiation of rituximab treatment in generalised myasthenia gravis**

### **1.1 Use of rituximab in generalised myasthenia gravis**

B-cell-directed therapy plays an important role in the management of gMG, despite the lack of formal regulatory approval. Among these agents, the CD20-inhibitor RTX has been widely used, and more recently the CD19-inhibitor inebilizumab has demonstrated positive results in the phase 3 MINT trial. ^1^ Despite an initial absence of randomised controlled trials (RCTs), RTX has become an established treatment over the past two decades, largely supported by case series, meta-analyses, and extensive clinical experience. ^2-7^ In AChR-Ab-positive MG, the BEAT-MG trial showed no advantage in refractory disease, whereas the RINOMAX trial demonstrated efficacy in new-onset MG – findings that have led to first-line recommendations for RTX in Scandinavian guidelines. ^2,3,8^ Beyond these two phase 2 trials and compared with newer targeted therapies such as FcRn-I and C5-I, however, the evidence base for rituximab remains less robust and phase 3 trials are still lacking. In MuSK-Ab-positive gMG, retrospective studies consistently demonstrate marked clinical benefit, with many patients achieving minimal manifestation status. ^9-11^ Overall, RTX is regarded as a valuable intensified immunotherapy in AChR-Ab-positive, MuSK-Ab-positive, and even seronegative MG, typically serving as an alternative to conventional steroid-sparing agents such as AZA or MMF. ^2-8,12^

**Expert consensus**

Recommendation (majority vote by 62.5%): RTX may be considered early after disease onset in patients with MuSK-Ab positive gMG when symptomatic therapy alone is insufficient. RTX should be considered as the first immunomodulatory monotherapy, irrespective of temporary corticosteroid use or crisis therapy in acute situations. Note: The use of RTX for this indication is off-label. Rozanolixizumab is approved as add-on therapy to standard treatment in this indication.

Recommendation (majority vote by 68.75%): RTX monotherapy may be considered early in patients with highly active gMG who are AChR-Ab-positive, LRP4-Ab-positive, or seronegative, as an alternative to standard steroid-sparing immunotherapies. **RTX should preferably be initiated soon after disease onset,** irrespective of temporary corticosteroid use or crisis therapy in acute situations. RTX may also be considered early in patients with comorbid autoimmune diseases for which RTX is already indicated (e.g. rheumatoid arthritis). Note: The use of RTX for gMG as a sole indication is off label.

Recommendation (consensus): RTX should be used as an intensified immunotherapy in addition to standard steroid-sparing agents in patients with AChR-Ab-positive, LRP4-Ab-positive, or seronegative highly active gMG who show an inadequate response to standard immunotherapies. Dosing should be determined individually, considering pathophysiology, prior therapies, and overall clinical condition, with careful assessment of risks and benefits. Note: The use of RTX for this indication is off label.

### **1.2 Safety considerations for rituximab therapy**

RTX is generally well tolerated. However, with repeated administration, rare but clinically relevant risks include infections – particularly opportunistic infections – associated with immunosuppression, and the potential development of hypogammaglobulinaemia. With a clinical response to RTX, concomitant steroid-sparing immunotherapies may be tapered and, if feasible, discontinued. The combination of RTX with standard immunotherapies carries an increased risk of progressive multifocal leukoencephalopathy (PML); therefore, such regimens remain controversial and treatment decisions should be made on an individual basis. ^12-14^

**Expert consensus**

Recommendation (consensus): Vaccination status should be verified prior to initiating RTX therapy, and any outstanding vaccinations (in accordance with national recommendations) should be updated with a minimum interval of two to four weeks between the last vaccination and the first RTX administration. Meningococcal vaccination should also be administered in advance of a potential future requirement for C5-I treatment. If the clinical condition does not allow immunisation during the acute phase, vaccinations should be performed after B-cell reconstitution (preferably not until 4–6 months after the last RTX cycle). Note: This recommendation can also be applied to other vaccinations but only refers to active immunization.

Recommendation (consensus): Screening for hepatitis B, hepatitis C, and tuberculosis (quantiferon test) infections should be performed prior to initiating RTX therapy. In patients with a history of hepatitis B infection, RTX therapy should either be avoided or antiviral prophylaxis—such as tenofovir disoproxil fumarate or entecavir—should be administered to prevent potential hepatitis B reactivation during RTX treatment. In the case of active HCV infection, RTX therapy should be avoided or postponed until successful treatment with direct-acting antiviral drugs has been completed. In patients with active tuberculosis, RTX therapy must be avoided. In those with latent tuberculosis, RTX therapy should only be initiated after appropriate prophylactic treatment.

Recommendation (consensus): B-cell counts (CD19/20) and, in particular IgG levels should be monitored during prolonged RTX therapy, as B-cell depletion may lead to hypogammaglobulinemia.

### **1.3 Dosing regimens and administration of rituximab**

In clinical practice, dosing regimens vary widely, reflecting pathophysiological considerations, local experience and established practice in related indications. ^3,8,14-16^ RTX can be initiated immediately after IA or plasma exchange in crisis situations. Based on the RINOMAX trial, a clinical response to RTX can be expected after approximately 4 months in patients with new-onset AChR-Ab-positive gMG. ^3^ In MuSK-Ab-positive MG, clinical improvement often occurs earlier, ^10,17^ whereas in long-standing AChR-Ab-positive or seronegative gMG, clinical benefit may take considerably longer to manifest. ^5,18^

In MuSK-Ab-positive gMG, the therapeutic effect frequently persists for several years, while in AChR-Ab-positive and seronegative gMG, efficacy usually last for six to 12 months. ^9^ Retreatment is typically performed at the end of the effective period – often at fixed 6- to 12-month intervals during the initial year – followed by gradual dose reduction or interval extensions. In the absence of clinical benefit, RTX is usually discontinued. When effective, the need for continued therapy should be reassessed regularly, aiming for the lowest possible dose and the longest feasible dosing intervals, including treatment pauses when appropriate. ^5^ Rising MuSK-Ab titers may serve as an early marker of relapse, while the reappearance of B cells – particularly CD19⁺CD20⁺ memory B cells – can guide retreatment decisions. ^19^

**Expert consensus**

Recommendation (consensus): Patients with MuSK-antibody-positive MG who previously responded to RTX should receive an additional RTX cycle at the first clinical signs of recurrence, which often occur even several years after initial therapy. Note: The use of RTX for this indication is off label.

Recommendation (consensus): In patients with highly active, AChR-antibody-positive generalised MG, including treatment-refractory cases, who receive early RTX as part of extended immunotherapy, further RTX cycles should be considered either at the first clinical signs of relapse or, depending on the clinical context, at predefined intervals (six to 12 months). At such intervals, the continued need for treatment should be reassessed by exploring longer dosing intervals or introducing treatment pauses. In cases of repeated administration, lower doses than those used for the initial treatment should also be considered. Note: The use of RTX for this indication is off label.

## **Chapter 2: Role of intravenous and subcutaneous immunoglobulins in generalised myasthenia gravis**

IVIg are approved and recommended for the treatment of severe exacerbations and (impending) myasthenic crisis in gMG, serving as an alternative to PLEX and IA. ^12,20^ In contrast, SCIg are not approved for MG. ^12^ The role of interval IVIg or SCIg therapy remains uncertain due to limited evidence, ^21,22^ while PLEX and IA are unsuitable for long-term use.

**Expert consensus**

Interval IVIg therapy should be considered as the treatment of choice under the following conditions:

Recommendation (strong consensus): When standard therapy (symptomatic treatment, oral steroid-sparing immunomodulators, glucocorticosteroids, thymectomy) and, where appropriate, FcRn-I or C5-I are insufficient to control the disease, or when absolute contraindications exist (e.g. immunodeficiency syndromes).

Recommendation (strong consensus): In the rare constellation where weaning from oral steroid-sparing immunomodulators and glucocorticosteroids is not possible, including during PLEX or IA in the intensive care setting for myasthenic crisis.

Recommendation (consensus): In patients who show clinical improvement with IVIg rescue therapy but experience recurrent deterioration after its discontinuation.

Recommendation (strong consensus): In specific situations that may precipitate severe exacerbations, such as pregnancy or family planning, the peripartum period, infections, or perioperative settings.

Note: The use of IVIG for interval therapy is off label for gMG.

The necessity of measuring serum IgA levels before initiating IVIG remains controversial. While some advocate baseline testing to identify patients with absolute IgA deficiency and potential risk of severe reactions, others regard routine screening as unnecessary given the rarity of clinically relevant IgA deficiency and the overall good tolerability of IVIg. Testing for anti-IgA antibodies is likewise not routinely performed and is mainly reserved for patients who experience severe infusion reactions. In patients at risk for thrombosis (e.g., those with a history of deep vein thrombosis, immobilisation, high-dose corticosteroids, or pregnancy), appropriate thrombosis prophylaxis is recommended. ^23^

IVIg is the preferred route of administration for initiation. IVIg is typically used first in MG because it achieves rapid systemic immunomodulation and is effective in acute or worsening disease states. SCIg, by contrast, is generally introduced later as a maintenance strategy, once patients are clinically stable and immediate high-peak immunoglobulin levels are no longer required. Treatment typically consists of a total dose of 2 g/kg body weight administered over two to five consecutive days, with daily doses of 30–50 g (maximum 80 g; < 40 g in multimorbid or high-risk patients). ^24,25^ Clinical improvement is usually observed within five to 14 days. ^26,27^

Caution is warranted in patients with cardiac or renal disease; in such cases, lower infusion rates, minimal effective dosing, and sucrose-free preparations are recommended. ^28,29^ In interval therapy, infusion intervals (typically four to 12 weeks) should be individualised according to clinical status, for example guided by MG-ADL scores.

Switching to SCIg may be considered in clinically stable patients with a documented and predictable duration of IVIg effect. Conversion from IVIG to SCIg is typically guided by the prior treatment interval, with total dosing often increased by 10–20% to achieve comparable clinical efficacy. AEs – such as flu-like symptoms, rash, or aseptic meningitis – are usually dose- or rate-dependent and may vary between preparations; switching product or formulation may alleviate these reactions. ^28,30^

**Expert consensus**

Recommendation (strong consensus): Initiating immunoglobulin therapy with the intravenous route (IVIg) should be the preferred approach.

Recommendation (strong consensus): A switch to SCIg in clinically stable patients on interval IVIg therapy with a documented duration of effect of four to 12 weeks should be considered.
Note: The use of IVIG or SCIg for interval therapy is off label for gMG.

## ***References***

1.	Nowak RJ, Benatar M, Ciafaloni E, et al. A Phase 3 Trial of Inebilizumab in Generalized Myasthenia Gravis. *N Engl J Med*. 2025;392(23):2309-2320. doi:10.1056/NEJMoa2501561

2.	Nowak RJ, Coffey CS, Goldstein JM, et al. Phase 2 Trial of Rituximab in Acetylcholine Receptor Antibody-Positive Generalized Myasthenia Gravis: The BeatMG Study. *Neurology*. 2022;98(4):e376-e389. doi:10.1212/wnl.0000000000013121

3.	Piehl F, Eriksson-Dufva A, Budzianowska A, et al. Efficacy and Safety of Rituximab for New-Onset Generalized Myasthenia Gravis: The RINOMAX Randomized Clinical Trial. *JAMA Neurol*. 2022;79(11):1105-1112. doi:10.1001/jamaneurol.2022.2887

4.	Brauner S, Eriksson-Dufva A, Hietala MA, Frisell T, Press R, Piehl F. Comparison Between Rituximab Treatment for New-Onset Generalized Myasthenia Gravis and Refractory Generalized Myasthenia Gravis. *JAMA Neurol*. 2020;77(8):974-981. doi:10.1001/jamaneurol.2020.0851

5.	Vesperinas-Castro A, Cortés-Vicente E. Rituximab treatment in myasthenia gravis. *Front Neurol*. 2023;14:1275533. doi:10.3389/fneur.2023.1275533

6.	Tandan R, Hehir MK, 2nd, Waheed W, Howard DB. Rituximab treatment of myasthenia gravis: A systematic review. *Muscle Nerve*. 2017;56(2):185-196. doi:10.1002/mus.25597

7.	Di Stefano V, Lupica A, Rispoli MG, Di Muzio A, Brighina F, Rodolico C. Rituximab in AChR subtype of myasthenia gravis: systematic review. *J Neurol Neurosurg Psychiatry*. 2020;91(4):392-395. doi:10.1136/jnnp-2019-322606

8.	Gilhus NE, Andersen H, Andersen LK, et al. Generalized myasthenia gravis with acetylcholine receptor antibodies: A guidance for treatment. *Eur J Neurol*. 2024;31(5):e16229. doi:10.1111/ene.16229

9.	Vakrakou AG, Karachaliou E, Chroni E, et al. Immunotherapies in MuSK-positive Myasthenia Gravis; an IgG4 antibody-mediated disease. *Front Immunol*. 2023;14:1212757. doi:10.3389/fimmu.2023.1212757

10.	Hehir MK, Hobson-Webb LD, Benatar M, et al. Rituximab as treatment for anti-MuSK myasthenia gravis: Multicenter blinded prospective review. *Neurology*. 2017;89(10):1069-1077. doi:10.1212/wnl.0000000000004341

11.	Díaz-Manera J, Rojas-García R, Gallardo E, et al. Antibodies to AChR, MuSK and VGKC in a patient with myasthenia gravis and Morvan's syndrome. *Nat Clin Pract Neurol*. 2007;3(7):405-10. doi:10.1038/ncpneuro0526

12.	Wiendl H, Abicht A, Chan A, et al. Guideline for the management of myasthenic syndromes. *Ther Adv Neurol Disord*. 2023;16:17562864231213240. doi:10.1177/17562864231213240

13.	Wagner N, Assmus F, Arendt G, et al. Impfen bei Immundefizienz : Anwendungshinweise zu den von der Ständigen Impfkommission empfohlenen Impfungen. (IV) Impfen bei Autoimmunkrankheiten, bei anderen chronisch-entzündlichen Erkrankungen und unter immunmodulatorischer Therapie *Bundesgesundheitsblatt Gesundheitsforschung Gesundheitsschutz*. 2019;62(4):494-515. Impfen bei Immundefizienz : Anwendungshinweise zu den von der Ständigen Impfkommission empfohlenen Impfungen. (IV) Impfen bei Autoimmunkrankheiten, bei anderen chronisch-entzündlichen Erkrankungen und unter immunmodulatorischer Therapie. doi:10.1007/s00103-019-02905-1

14.	European Medicines Agency. MabThera, Rituximab. Summary of product characteristics. <https://www.ema.europa.eu/en/documents/product-information/mabthera-epar-product-information_en.pdf> (last accessed: 09/23/2025).

15.	Du Y, Li C, Hao YF, et al. Individualized regimen of low-dose rituximab monotherapy for new-onset AChR-positive generalized myasthenia gravis. *J Neurol*. 2022;269(8):4229-4240. doi:10.1007/s00415-022-11048-4

16.	Héraud C, Bresch S, Landes-Château C, Bourg V, Lebrun-Frenay C. Rituximab alone is as effective as associated with steroids on naive patients with generalized myasthenia gravis. *J Neurol*. 2024;271(8):5197-5202. doi:10.1007/s00415-024-12454-6

17.	Chayanopparat S, Banyatcharoen P, Jitprapaikulsan J, Uawithya E, Apiraksattayakul N, Viarasilpa V. Efficacy and safety of rituximab in anti-MuSK myasthenia Gravis: a systematic review and meta-analysis. *Sci Rep*. 2025;15(1):7219. doi:10.1038/s41598-025-90937-w

18.	Litchman T, Roy B, Kumar A, Sharma A, Njike V, Nowak RJ. Differential response to rituximab in anti-AChR and anti-MuSK positive myasthenia gravis patients: a single-center retrospective study. *J Neurol Sci*. 2020;411:116690. doi:10.1016/j.jns.2020.116690

19.	Fichtner ML, Hoehn KB, Ford EE, et al. Reemergence of pathogenic, autoantibody-producing B cell clones in myasthenia gravis following B cell depletion therapy. *Acta Neuropathol Commun*. 2022;10(1):154. doi:10.1186/s40478-022-01454-0

20.	Camps F, Sander HW, Fernández S. Intravenous immunoglobulin (Gamunex® 10%) is licensed for the treatment of severe acute exacerbations of myasthenia gravis. *Expert Opin Pharmacother*. 2023;24(5):665. doi:10.1080/14656566.2023.2192867

21.	Alzuabi MA, Manolopoulos A, Elmashala A, Odabashian R, Naddaf E, Murad MH. Immunoglobulin for myasthenia gravis. *Cochrane Database of Systematic Reviews*. 2020;(12)doi:10.1002/14651858.CD013801

22.	Gajdos P, Chevret S, Toyka KV. Intravenous immunoglobulin for myasthenia gravis. *Cochrane Database of Systematic Reviews*. 2012;(12)doi:10.1002/14651858.CD002277.pub4

23.	Guo Y, Tian X, Wang X, Xiao Z. Adverse Effects of Immunoglobulin Therapy. *Front Immunol*. 2018;9:1299. doi:10.3389/fimmu.2018.01299

24.	Ruzhansky K, Brannagan TH, 3rd. Intravenous immunoglobulin for treatment of neuromuscular disease. *Neurol Clin Pract*. 2013;3(5):440-445. doi:10.1212/CPJ.0b013e3182a78ecf

25.	Farmakidis C, Pasnoor M, Dimachkie MM, Barohn RJ. Treatment of Myasthenia Gravis. *Neurol Clin*. 2018;36(2):311-337. doi:10.1016/j.ncl.2018.01.011

26.	Evoli A, Palmisani MT, Bartoccioni E, Padua L, Tonali P. High-dose intravenous immunoglobulin in myasthenia gravis. *Ital J Neurol Sci*. 1993;14(3):233-7. doi:10.1007/bf02335664

27.	Achiron A, Barak Y, Miron S, Sarova-Pinhas I. Immunoglobulin treatment in refractory Myasthenia gravis. *Muscle Nerve*. 2000;23(4):551-5. doi:10.1002/(sici)1097-4598(200004)23:4<551::aid-mus14>3.0.co;2-o

28.	Stiehm ER. Adverse effects of human immunoglobulin therapy. *Transfus Med Rev*. 2013;27(3):171-8. doi:10.1016/j.tmrv.2013.05.004

29.	Pierce LR, Jain N. Risks associated with the use of intravenous immunoglobulin. *Transfus Med Rev*. 2003;17(4):241-51. doi:10.1016/s0887-7963(03)00038-5

30.	Kareva L, Mironska K, Stavric K, Hasani A. Adverse Reactions to Intravenous Immunoglobulins - Our Experience. *Open Access Maced J Med Sci*. 2018;6(12):2359-2362. doi:10.3889/oamjms.2018.513
